# Early Postnatal Care Service Utilization and Its Determinants among Women Who Gave Birth in the Last 6 Months in Wonago District, South Ethiopia: A Community-Based Cross-Sectional Study

**DOI:** 10.1155/2021/4286803

**Published:** 2021-04-14

**Authors:** Yordanos Tefera, Samirawit Hailu, Ruth Tilahun

**Affiliations:** ^1^Department of Public Health, College of Health & Medical Science, Dilla University, Dilla, Ethiopia; ^2^Department of Midwifery, College of Health & Medical Science, Dilla University, Dilla, Ethiopia

## Abstract

**Background:**

Postnatal care is one of the key strategies to reduce maternal and newborn morbidity and mortality. Early postnatal visit is especially the most critical time for survival of mothers and newborns, particularly through early detection and management of postpartum complication. Despite the benefits, most mothers and newborns do not receive postnatal care services from health care providers during the critical first few days after delivery.

**Objective:**

The aim of this study was to assess utilization of early postnatal care service and associated factors among women who gave birth in the last six months in Wonago District, Gedeo Zone, Southern Ethiopia.

**Methods:**

A community-based cross-sectional study design was employed at Wonago District. A total of 612 mothers who gave birth in the last six months were selected by simple random sampling technique. Pretested structured questionnaire was used for data collection. Data were entered into EpiData version 3.1 and then exported into SPSS version 20 for analysis. Principal component analysis (PCA) and bivariate and multivariate logistic regression were used.

**Result:**

In this study, 13.7% of mothers utilized early postnatal care. Educational status of mothers (AOR = 3.7 : 95 CI; 1.3–10.7), place of delivery (AOR: 1.8 : 95 CI; 1.03–3.2), ANC attendance (AOR = 3.4 : 95 CI; 1.1–10.09), development of complication after delivery (AOR: 7.8 : 95 CI; 3.7–16.2), and previous history of postnatal care utilization (AOR: 2.1 : 95 CI; 1.13–3.9) were found to be associated with early postnatal care service utilization. *Conclusion and Recommendations*. Educational status of mothers, ANC attendance, place of delivery, delivery complication while giving recent birth, and past history of postnatal care utilization were significant predictors for early postnatal care utilization. Considering this, empowering women with education and overall strengthening of health facility to improve maternal health service utilization are necessary measures to be done at different levels to enhance early postnatal care utilization during this critical time.

## 1. Background

Postnatal care (PNC) is the care given to the mother and her newborn baby immediately after birth of baby up to the first six weeks of life, and it is decisive to the general wellbeing of both the mother and the newborn [1]. Three phases of contact are recommended which include the immediate postnatal period within the first 24 hours after birth, early postnatal period from day two through 7, and late postnatal period extending from day 8 through 42 days [2].

PNC visit is very critical time for the survival of both mother and child. The early postnatal period, especially the first week after delivery, is the time when effective postnatal care can bring a difference to the health and life chances of mothers and newborns [3]. Since early postnatal care is very essential service to protect the mother from birth-related complications, much attention should be given by health care providers [4].

A large proportion of maternal and neonatal deaths occur during the 48 hours after delivery. Every year in Africa, at least 125,000 women and 870,000 newborns die in the first week after birth. It has been estimated that, 10 to 27 percent of newborn deaths could be averted if routine postnatal and curative care in the postnatal period reached 90 percent of babies and their mothers, which means that high PNC coverage could save up to 310,000 newborn lives each year in sub-Saharan Africa [5].

Despite the fact that early postnatal care visits are one the most important maternal health care interventions for prevention of morbidity and mortality, globally, only 48% of mothers are following the postnatal follow-up within two days of childbirth [5]. In developed countries, nearly all women and their infants receive postpartum and postnatal care. In developing countries, the need for care and support after birth was, until recently, less well recognized [2].

Evidence indicates that, in developing countries, proportion of women who had at least one postnatal care utilization within 42 days of delivery was 36% [6]. Every day, 800 women die from maternal causes. Almost all of these deaths (99%) occur in developing countries. One of the major indicators of the difference between developed and underdeveloped countries is maternal mortality. But this could easily be avoided as the necessary medical interventions exist and are well known. The key obstacle is the lack of access to quality care by pregnant women before, during, and after childbirth [7].

Most maternal and infant deaths occur in the first month after birth, almost half of postnatal maternal deaths occur within the first 24 hours, and 66% occur during the first week. 2.8 million newborns died in their first month of life; 1 million of these newborns died on the first day [8].

Higher number of maternal and newborn deaths occurs in the first week, especially on the first day. Early visits play a significant role in the prevention and management of many life threatening maternal and neonatal complications. This period is also the key time to promote healthy behaviors affecting women, newborns, and children, but the period following birth in Africa is often marked by cultural practices. Low coverage of care in the postnatal period negatively influences other maternal, newborn, and child health (MNCH) programs [9].

Approximately one-third of women in sub-Saharan Africa give birth in facilities, and no more than 13 percent of women who gave birth at home received a postnatal care visit within two days of delivery [10].

The 2016 Ethiopia Demographic Health Survey (EDHS) report stated that PNC service utilization is very much lower in Ethiopia; only 17% of women had received PNC during the first 2 days after birth. The proportion of women who received postnatal check-ups in the 2 days after delivery was 16.9*%* SNNPR [11]. However, there is limited information about current utilization of postnatal care service in the study area. Therefore, this study is vital to visualize level of early PNC utilization and serve as an important tool for any possible interventions.

## 2. Methods

### 2.1. Study Area and Period

The study was conducted in Wonago District, which is one of the 6 districts in Gedeo Zone, SNNPRS (Southern Nations, Nationalities and Peoples Regional State) and Ethiopia. The district is found 377 km south of the capital city Addis Ababa and 102 km from the region capital Hawassa. The district has 21 kebeles (17 rural and 4 urban) and hosts a total population of 156,480 within 30,442 households. The dominant ethnic group in the district is Gedeo. Six health centers, 20 health posts, and 2 private clinics provide the overall health care service in the districts. The total population of districts is 149,165. This study was conducted from December 5, 2017, to January 18, 2018.

### 2.2. Study Design and Population

Community-based cross-sectional study was conducted among randomly selected mothers who gave birth in last six months preceding the data collection. Mentally and/or physically incapable mothers and those not living in the last six months in Wonago District were excluded from the study.

### 2.3. Sample Size Determination and Sampling Technique

The minimum required sample size of this study was determined using single population proportion formula. Proportion of postnatal care service utilization [12], margin of error, confidence interval, design effect, and nonresponse rate were assumed to be 23.7%, 5%, 95%, 2%, and 10%, respectively:

(1) $$\begin{array}{c} n=\frac{\left({\left(Z\alpha \right)}^{2}/2\right)*P\left(1-P\right)}{d^{2}}\\ n=\frac{{\left(1.96\right)}^{2}*0.237\left(1-0.237\right)}{{\left(0.05\right)}^{2}}277*2=556. \end{array}$$

Then, adding 10% nonresponse rates resulted in the total sample required for the study being 612.

Stratified sampling technique was used in order to obtain a representative sample. The kebeles were stratified into urban (2 kebeles) and rural (19 kebeles) settings then 8 kebeles (two from urban kebeles and 6 from rural kebeles) were selected randomly from the total 21 kebeles in the district. In each selected kebele, sampling frame which comprises a list of mothers who gave birth in the last six months and their full address was prepared with the help of HEW from health post registrations book (family folders).

From this sampling frame, a total sample size of 612 which was distributed based on proportional size allocation or proportional to the number of mothers who gave birth in the last six months prior to study in each kebele was traced to participate in the study. Finally, the study units (mothers who gave birth in last six months) in each HH were selected by random sampling technique.

### 2.4. Data Collection Tool and Procedure

Data were collected using structured questionnaires adapted and modified after reviewing different literature as appropriate so as to address the study objectives [13–15].

The main contents of questionnaire were sociodemographic characteristics, obstetric factor, maternal knowledge on PNC and danger sign on postpartum period, and health service-related factors.

Early postnatal care service utilization was measured as mothers who received at least one PNC service starting immediately after time of delivery up to end of first week. The data were collected by trained data collectors who had diploma in health-related fields and who were fluent in Gedeo-Offa and Amharic.

The questionnaire was carefully designed and prepared in English language first and then translated into local languages (Gedeo-Offa) and Amharic. To check for consistency, the questionnaire was further translated from the local languages (Gedeo-Offa) and Amharic to English. Test-retest reliability was measured to determine the reliability of the tool and to measure its internal consistency; Cronbach's alpha was used, and it becomes 0.7.

### 2.5. Data Quality Control

To assure the data quality, one-day training was given for data collectors and supervision was made at the time of data collection in each kebele; also, data collection tools were translated from English to Amharic and Gedeo-Offa by experts and backtranslated to English to check the consistency. Moreover, a pretest was done in Harsu kebele by taking 5% of the total sample size before one week of the actual data collection, and data were checked for completeness and corrective measures were taken immediately.

### 2.6. Data Processing and Analysis

Data were cleaned and entered using EpiData version 3.1 and then exported to SPSS version 20 for analysis. Data were analyzed using SPSS version 20. Descriptive statistics were computed. Binary and multivariate logistic regression analyses were done to see the association between the dependent and independent variables. Binary logistic regression was used to identify variables that are a candidate for multivariate logistic regression analysis at *p* value < 0.25, and multivariate logistic regression analysis was used to determine the factors that are independently associated with early postnatal care utilization at *p* value < 0.05 with a 95% confidence level. Finally, variables with *p* value < 0.05 were considered statistically significant. Model fitness was assessed through the Hosmer and Lemeshow test (*p* = 0.756).

### 2.7. Variables of the Study

#### 2.7.1. Dependent Variable

The dependent variable is early postnatal care utilization.

#### 2.7.2. Independent Variable

- *Sociodemographic characteristics:* age, education, marital status, residence, occupation, education and occupation of the partner, and wealth index.
- *Obstetric factors:* gravidity, parity, birth order, ANC utilization, number of ANC visits, PNC utilization, timing of PNC visit, place for PNC, *frequency* of PNC, delivery complication after recent birth, and previous history of PNC.
- *Maternal knowledge on PNC:* knowledge availability and benefits of PNC service, knowledge on danger sign of postpartum period, and knowledge on maternal and neonatal care on postpartum period.
- *Health service-related factor:* time to reach health facility, advice/information provided by health care provider on postpartum danger sign, appointment for PNC during discharge, place of delivery, mode of delivery, and visit by HEW at home.

### 2.8. Operational Definitions

*Early postnatal care utilization* refers to mothers or their newborn babies who had at least one early postnatal care check-up for the current delivery by health care provider and health extension workers within first week of delivery.

### 2.9. Ethical Considerations

Ethical clearance to conduct this study was obtained from Dilla University, College of Health Science and Medicine Institutional Review Board. Written consent from each subject was asked and secured after detailed explanation of the main purpose of the study. All the provided information was strictly confidential, and written consent was taken. The participants had the right to withdraw from study at any time they want. The name of the respondent was not appearing on the questionnaire.

## 3. Results

### 3.1. Sociodemographic Characteristics of Mothers

The study included a total of 612 mothers who gave birth six months prior to the study with a response rate of 100%. The mean age of mothers was 26.2 years (SD = 4.9). 277 (45.3%) were within the age group of 20–26 years. Almost all of the respondents, 600 (98%), were married. About 477 (77.9%) were living in rural area. Regarding educational status, more than half of the mothers, 348 (56.9%), did not attend formal education and 512 (83.7%) of them were housewives (Table 1).

### 3.2. Obstetric Characteristics of Mothers

Above half, 402 (65.7%), of study participants had gravidity of 2–4 and most mothers had two or more babies (multiparas). From the total of respondents, 483 (78.9 %) of the mothers had antenatal care follow-up during recent pregnancy (Table 2).

### 3.3. Knowledge of Mothers about PNC Service Utilization

**Figure 1:**
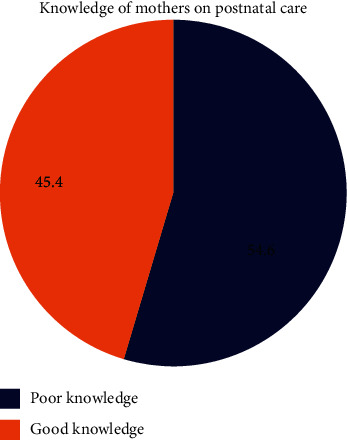
Knowledge about postnatal care utilization among mothers who gave birth in the last six months at Wonago District, South Ethiopia, 2017.

Out of the total respondents, 465 (75.9%) of mothers had ever heard of availability and importance of PNC services after delivery. About 54.6% of them had good knowledge of PPP (Figure 1).

### 3.4. Utilization of Early Postnatal Care

Of all respondents, about 184 (30.1%, 95 % CI, 26.4%–33.7%) mothers had used postnatal care services after recent delivery and the level of early postnatal care service utilization was only 84 (13.7%, 95% CI, 10.9%–16.4%) of the 612 participants. Among mothers who got postnatal care service, almost all of the respondents stated that they got postnatal care services one time, and most, 112 (60.3%), of postnatal care services users had got services at health center (Table 3).

### 3.5. Reason for Not Attending Early Postnatal Care Service

**Figure 2:**
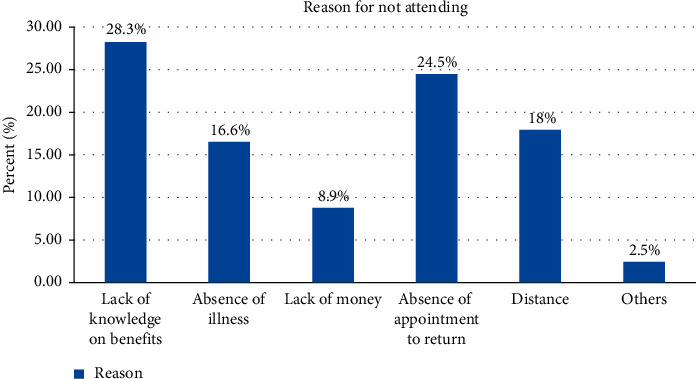
Respondents' reasons for not attending early postnatal care service among mothers who gave birth in the last six months at Wonago District, South Ethiopia, 2017.

Absence of illness immediately after delivery and lack of knowledge on the benefits of early postnatal care service utilization were the most raised reasons for not utilizing early postnatal care service (Figure 2).

### 3.6. Early Postnatal Care Service Utilization and Health Service-Related Factors

From the responses, most of the respondents travel more than 30 minutes to reach the health facility (252 (41.2%)); 353 (57.7%) gave birth in health center, from them only 129 (49.8%) got an appointment for PNC, and only 137 (22.4%) had got PNC service by health extension workers at their home (Table 4).

### 3.7. Factors Associated with Early Postnatal Care Service Utilization

All variables were assessed independently with the dependent variable in the bivariate logistic regression analysis, and those variables having a *p* value < 0.25 were candidates for a multivariate logistic regression analysis (see Table 5). Then from multivariate logistic regression analysis at *p* < 0.055, educational status of mothers (AOR: 3.7; 95% CI: 1.3, 10.6), place of delivery in the health institution (AOR 1.8, 95% CI: 1, 3.2), experience of PNC utilization for previous child (AOR: 2.10; 95% CI: 1.1, 3.9), ANC attendance before giving last birth, and having complication after giving recent birth were found to be statistically significant predictors for early postnatal care utilization.

The Hosmer–Lemeshow goodness of fit test (*p*=0.085) provides evidence of model fitness with the predictors.

Those mothers who attended above secondary school were 3.7 times more likely to utilize the service than those who did not attend formal education (AOR: 3.7; 95% CI: 1.3, 10.6).

Moreover, mothers who gave their last birth at health institution (AOR 1.8, 95% CI: 1, 3.2) were two times more likely to got early postnatal care service utilization when compared to those mothers who gave birth at home.

Similarly, mothers who have at least one ANC attendance before giving their last birth were 3.4 times (AOR: 3.4, 95% CI: 1.1, 10) more likely to utilize early service than those mothers who have no history of ANC attendance at all.

Furthermore, mothers who develop complication after giving recent birth were 7.8 times more likely to get early postnatal care services utilization than mothers who did not develop complication after giving birth (AOR: 7.8, 95% CI: 3.7, 16.2).

Likewise, previous experience of PNC has also been one of the associated factors with early PNC service utilization. Mothers with experience of postnatal care utilization for previous children were 2 times more likely to utilize early postnatal care than mothers who had no experience of postnatal care for previous child (AOR: 2.10; 95% CI: 1.1, 3.9) (see Table 5).

## 4. Discussion

The study revealed that, among 612 postpartum mothers, the level of early PNC service utilization was 84 (13.7%, 95% CI, 10.9%–16.4%). This implies that substantial proportion of postpartum mothers did not use early PNC.

Proportion of mothers that used early PNC service in this study was much lower as compared with the finding in Nepal, where 43.2% of the mothers had attended immediate PNC [16]. This difference in service provision attributed to differences in socioeconomic status, geographical barriers, and accessibility of service, information, and education between countries.

Moreover, community-based cross-sectional study done in Debre Markos town in Ethiopia in 2015 revealed a higher figure which shows that 60.4% of mothers had got postnatal care services within 3–7 days following birth and in Arsis zone, 23.7% of mothers had attended early postnatal care service within one week of delivery [12, 17]. This discrepancy might be because mothers of these study areas were less educated and less aware of the importance of postnatal care service utilization. In addition, it also might be due to difference in accessibility and availability of health service in urban area, since the majority of study participants lived in rural setting.

However, this study showed the early postnatal service utilization in the district to be slightly higher than the case of Mundri East Country, in South Sudan, where 11.4% of women used early PNC and 11.9% in Tigray, 2014 [18, 19]. The attribute might be due to the study time period difference reported by Tigray and in this study there could be improvement in accessing and utilizing health care service through time and other factors attributed to place and social context variation between this study and previous studies. In addition, it might be also variation in study area and study subjects.

Mothers who attended secondary and above education were 3.7 times more likely to attend early PNC service utilization as compared with those who did not attend formal education. The finding is consistent with studies conducted in Nepal, Pakistan, Morocco, Kenya, Tanzania, South Sudan, and in Amhara region, Ethiopia [16, 19–22]. In addition, it is supported by local study conducted in Jebetina District, Amhara region, and study conducted in Abi-Adi Town, Tigray region [18, 23]. This shows that education has a valuable input in order to empower women to gain access to health promotion message and information on how to obtain service and importance of available services. This is because educated mothers have an opportunity to participate in different social and economic positions and social empowerment.

Mothers who gave birth to their last child at health institution had 80% increased odds of getting early postnatal care service utilization when compared with mothers who gave birth to their last child at home. The finding was consistent with studies from Nepal, Pakistan, Kenya, Nigeria, Uganda, and Tanzania [20–22, 24, 25]. In addition to this, the result was nearly consistent with evidence from local finding in Jebitena District, Debre Markos Town, Lemo Woreda, Wolayita Zone, and Arsi Zone [12, 17, 23, 26, 27]. The explanation for this association was attributed to the fact that women who gave their last birth in health institution have greater opportunity to get exposed to health education and advice or counseling related to PNC service at the time of delivery. Besides this, they can get access to learn about the types, benefits, and availabilities of PNC services from skilled attendants during their stay in the health institutions.

Similarly, utilization of ANC has been found to positively influence early PNC service utilization. Mothers who had at least one attendance of ANC were more likely to utilize early PNC service than mothers who did not have ANC at all. This result was supported by the studies conducted in Gondar Zuria Districts, Amhara regions [28], Lemo Woreda, [27], Nepal [25], Benin, and Pakistan [22]. This is because mothers will have the opportunity to receive health education and counseling from health professionals about availability and benefit of PNC and schedules during their ANC follow-up. Attending ANC provides pregnant women the chance to obtain necessary health information on possible preparation for childbirth and advantage of delivering at health facility, and they generally get access to learn about the importance of utilization PNC service along with ANC examination.

Mothers who faced delivery complication after giving last birth were 7.8 times more likely to get early postnatal care services than mothers who did not face any complication after giving birth (AOR: 7.8, CI: 3.9–16.5). The finding agreed with the result of studies done in Debre Markos, Hadiya Zone, and Nepal in 2013 [17, 25, 27]. It is explained by the fact that exposure of complication increases fear of additional health complication; mothers who had experienced some danger sign were more likely to have increased perception of susceptibility and severity of the risks of maternal morbidity and mortality and which resulted in increased utilization of early postnatal service.

Mothers with experience of postnatal care for previous child were 2 times more likely to utilize early postnatal care than mothers who had no experience. This finding was consistent with cross-sectional study done in Gondar Zuria, in Addis Ababa town, and in Arsi zone [12, 15, 28]. This could be explained by the fact that experienced mothers had a better opportunity to get information/advice provision on the importance of early postnatal care service follow-up for both mother and newborn during previous childbirth from health care providers.

## 5. Conclusion

Less than one-fifth of the participants were using early postnatal care service. This figure was low as compared with most of the studies conducted in other parts of Ethiopia and other countries. Educational status of the mothers, place of delivery, delivery complication, and previous history of postnatal care were found to be significantly associated with early postnatal care service utilization.

## Figures and Tables

**Table 1:** Sociodemographic characteristics of mothers who gave birth in the last six months at Wonago District, South Ethiopia, 2017 (*n* = 612).

Variables	Frequency	Percent (%)
*Age of mother at interval*		
≤19	21	3.4
20–26	277	45.3
27–32	252	41.2
≥33	62	10.1

*Marital status of mothers*		
Married	600	98
Others	12	2

*Place of residence*		
Rural	508	83
Urban	104	16.9

*Educational status of mother*		
No formal education	348	56.9
Primary	217	35.5
Secondary and above	47	7.6

*Educational status of father*		
No formal education	151	25.2
Primary	328	54.6
Secondary and above	121	20.2

*Occupational status of mother*		
House wife	512	83.7
Farmer	5	0.8
Daily labourer	19	3.1
Government employee	14	2.3
Merchant	62	10.1

*Occupational status of father*		
Farmer	290	48.3
Daily labourer	96	16
Government employee	39	6.5
Merchant	175	29.2

*Wealth index*		
Low	205	33.5
Middle	203	32.2
High	204	33.3

**Table 2:** Obstetric characteristics of mothers at Wonago District, South Ethiopia, 2017 (*n* = 612).

*Variables*	Frequency	Percent (%)
*Number of pregnancies*		
One	82	13.4
Two–four	402	65.7
Five and above	128	20.9

*Number of alive births*		
One	82	13.4
Two–four	402	65.7
Five and above	127	20.8

*Birth order of last pregnancy*		
First	83	13.6
Second	111	18.1
Third	225	36.8
Four and above	193	31.5

*ANC visit*		
Yes	483	78.9
No	129	21.1

*Number of ANC visits (n* *=* *483)*		
≤2	109	22.5
≥3	374	77.5

*Past history of PNC*		
Yes	104	17
No	508	83

**Table 3:** Utilization of early postnatal care service among mothers who gave birth in the last six months at Wonago District, South Ethiopia, 2017.

*Variables*	Frequency	Percent (%)
*PNC visit (n* *=* *612)*		
Yes	184	30.1
No	428	69.9

*Time for PNC (n* *=* *184)*		
Within 24 hours	22	11.9
2-3 days	11	7.6
4–7 days	48	26
8–42 days	100	54.5

*Early PNC utilization (n* *=* *84)*		
Yes	84	13.7
No	528	86.3

*Frequency for PNC (n* *=* *184)*		
One	84	45.6
Two	78	42.3
Three and above	22	11.9

*Place for PNC (n* *=* *184)*		
Home	41	22.3
Health center	112	60.3
Clinic or health post	31	16.9

**Table 4:** Health service-related factors of respondents among mothers who gave birth in that last six months at Wonago District, South Ethiopia, 2017.

Variables	Frequency	Percent (%)
*Time to reach health facility (n* *=* *612)*		
<15 min	194	31.7
15–30	166	27.1
>30	252	41.2

*Place of delivery*		
Home	353	57.7
Health center	259	42.3

*Get appointment for PNC (N* *=* *259)*		
Yes	129	49.8
No	130	50.2

*Advise on postpartum danger sign (N* *=* *259)*		
Yes	116	44.8
No	143	55.2

*Visit by HEW at home (n* *=* *612)*		
Yes	137	22.4
No	475	77.6

**Table 5:** Multivariable analysis of factors associated with early PNC utilization of mothers at Wonago District, South Ethiopia, 2017.

Variables	Early PNC attendance		Crude	Adjusted
	No (%)	Yes (%)	OR (95% CI)	OR (95% CI)
*Mothers education*				
No formal education	317 (91.1)	31 (8.9)	1	1
Primary	183 (84.3)	34 (15.7)	1.9 (1.13–3.19)	1.37 (0.72–2.61)
Secondary and above	28 (59.9)	19 (40.4)	6.93 (3.48–13.82)	**3.77 (1.33–10.65)∗∗**

*Husband education*				
No formal education	136 (92.5)	11 (7.5)	1	1
Primary	291 (86.9)	44 (13.1)	1.86 (0.93–3.73)	1.51 (0.72–3.27)
Secondary and above	90 (76.3)	28 (23.7)	3.84 (1.82–8.11)	1.47 (0.56–3.85)

*Occupation of mother*				
House wife	451 (88.1)	61 (11.9)	1	1
Farmer	21 (87.5)	3 (12.5)	1.05 (0.30–3.64)	0.89 (0.23–3.44)
Government employee	9 (64.3)	5 (35.7)	4.10 (1.33–12.65)	1.64 (0.41–6.43)
Merchant	47 (75.8)	15 (24.2)	2.36 (1.24–4.47)	1.26 (0.58–2.77)

*Occupation of father*				
Farmer	261 (90.3)	28 (9.7)	1	1
Government employee	28 (73.3)	10 (26.3)	3.26 (1.44–7.39)	1.40 (0.45–4.31)
Merchant	141 (79.9)	36 (20.3)	2.25 (1.32–1.32)	1.77 (0.93–3.37)
Daily labourer	87 (90.6)	9 (9.4)	0.92 (0.42–2.02)	0.72 (0.29–1.73)

*Having ANC*				
No	125 (96.9)	4 (3.1)	1	1
Yes	403 (83.4)	80 (16.9)	6.2 (2.22–17.27)	**3.4 (1.15–10.09)∗**

*Number of alive births*				
One	67 (80.7)	16 (19.3)	1	1
Two–four	346 (86.6)	56 (13.9)	0.67 (0.36–1.25)	0.75 (0.21–2.74)
Five	115 (90.6)	12 (9.4)	0.43 (0.19–0.97)	1.01 (0.34–2.96)

*Birth order*				
First	67 (80.75)	16 (19.3)	1	1
Second	94 (84.7)	17 (15.3)	0.75 (0.35–1.60)	1.82 (0.39–8.46)
Third	190 (84.4)	35 (15.6)	0.77 (0.40–1.48)	2.45 (0.55–10.82)
Fourth and above	177 (91.7)	16 (8.3)	0.37 (0.17–0.80)	1.06 (0.36–3.12)

*Place of delivery*				
Home	326 (92.4)	27 (7.6)	1	1
Health center	202 (78)	57 (22)	3.4 (2.08–5.56)	**1.8 (1.03–3.26)∗**

*Delivery complication*				
No	504 (89.4)	60 (10.6)	1	1
Yes	24 (50)	24 (50)	8.4 (4.49–15.7)	**7.83 (3.76–16.28)∗∗**

*Past history of PNC*				
No	450 (88.6)	58 (11.4)	1	1
Yes	78 (75)	26 (25)	2.58 (1.53–4.35)	**2.10 (1.33–3.90)∗∗**

*HEW visit at home*				
No	420 (88.4)	55 (11.6)	1	1
Yes	108 (78.8)	29 (21.2)	2.05 (1.24–3.37)	0.77 (0.42–1.39)

CI: confidence interval; OR: odds ratio; ANC: antenatal care; PNC: postnatal care; 1: reference variable; ^*∗*^significant at *p* < 0.05; ^*∗∗*^significant at *p* < 0.01.

## Data Availability

The datasets used and/or analyzed during the current study are available from the corresponding author on reasonable request.
